# Predictive Risk Factors for Childbirth-Associated Breast Infections in the United States: A 10-Year Perspective

**DOI:** 10.3390/ijerph20146333

**Published:** 2023-07-10

**Authors:** Klaudia J. Koziol, Abbas Smiley, Rifat Latifi, Maria T. Castaldi

**Affiliations:** 1 School of Medicine, New York Medical College, Valhalla, NY 10595, USA; 2 Westchester Medical Center, Valhalla, NY 10595, USA

**Keywords:** mastitis, breast infection, breast abscess, risk factors, breastfeeding

## Abstract

Infectious mastitis is a common condition that affects up to 33% of lactating women. Several risk factors have been suggested to be strongly associated with breast abscess, nipple infection, and non-purulent mastitis associated with childbirth. In this retrospective cohort study, we gathered data from the National Inpatient Sample (NIS) between 2005 and 2014 and utilized data stratification and backward linear regression to analyze the predictive factors associated with patients hospitalized with breast infection after childbirth, with special consideration of risk factors affecting hospital length of stay (LOS). In the ten-year period, 4614 women were hospitalized with a primary diagnosis of breast abscess, nipple infection, or non-purulent mastitis associated with childbirth. Mean (SD) age was 26.75 (6) years. The highest frequency distribution of cases was observed in patients aged 22–30 years (49.82%). Mean (SD) LOS was 2.83 (1.95) days. Mean (SD) LOS in patients with procedure was 3.53 (2.47) days, which was significantly longer than that in those with no procedure (2.39 (1.36) days, *p* < 0.001). Primary diagnosis of breast abscess and occurrence of a hospital procedure were most significantly associated with prolonged LOS. Factors such as age, socioeconomic position, severity of functional loss, as well as comorbidities were also contributing risk factors to the development of breast infection and increased hospital LOS. Further studies should examine these findings, as they relate to breastfeeding practices and concentrate on establishing best practices for risk reduction and prevention of childbirth-associated breast and nipple infections and hospitalizations.

## 1. Introduction

Mastitis is most commonly encountered during the postpartum period and affects up to 33% of women during lactation [1]. Breastfeeding, offering both infant and maternal health benefits and recommended exclusively for at least six months by all major health organizations, including the World Health Organization (WHO) and the Centers for Disease Control (CDC), may be adversely affected by breast disease [1,2,3]. Infections of the breast, including nipple infections, mastitis, and breast abscesses, are common among breastfeeding women and can often lead to premature cessation of breastfeeding [1,4]. 

Mastitis is characterized by localized inflammation, swelling, and erythema of the breast, causing pain, tenderness, fever, and difficulty breastfeeding in the mother. Flu-like symptoms, malaise, axillary lymph node enlargement, and decreased milk outflow are also common symptoms [5,6]. The etiology of mastitis is multifactorial. Historically, it has been thought that milk stasis leads to a blockage of a milk duct, and cracks and fissures of the nipple, due to mechanical irritation from a poor latch or infant mouth anomalies, allow bacteria to gain entry from the infant’s mouth or mothers skin, which also contributes to the pathology of both mastitis as well as nipple infection [7]. However, new research has suggested that the pathophysiology may be much more complex and result from a combination predisposing and protecting host, microbial, and medical factors, which contribute to dysbiosis of the mammary gland [8]. *Staphylococcus aureus* is the most common pathogen, but infection can also be due to *Streptococcus pyogenes* as well as fungal species such as *Candida albicans* and *Mycobacterium tuberculosis* [4,5]. In cases of Staphylococcus aureus, it is critical to identify whether MRSA is involved, as the risk of progression to abscess is significantly increased [9]. The incidence of mastitis is 20% in breastfeeding women, although studies report a varying range from 2% up to 33% [4,10,11,12]. Less common sequelae of mastitis are breast abscesses, which are localized areas of purulent material within breast tissue [5,7]. Studies report 3% to 11% of women with mastitis develop an abscess, with a total incidence between 0.1% and 3% in all breastfeeding women overall [6].

Treatment recommendations for mastitis include continuation of breastfeeding, local cold compress to the breast, and antibiotics, while progression to breast abscesses may require a drainage procedure [5,10,13,14]. Several risk factors have been suggested to be strongly associated with breast abscess, nipple infection, and non-purulent mastitis associated with childbirth. In this article, our objective is to analyze the risk factors for such infections and hospitalizations, with special consideration of risk factors affecting hospital length of stay (LOS).

## 2. Materials and Methods

The National Inpatient Sample (NIS) is the largest publicly available all-payer inpatient care database in the United States and is a part of the Healthcare Cost and Utilization Project (HCUP), a collection of databases sponsored by the Agency for Healthcare Research and Quality (AHRQ). Using billing data on inpatient admissions submitted by hospitals nationwide, the database contains hospitalization data on more than seven million hospital stays annually. The large sample size is ideal for assessing national and regional estimates. The NIS database is available at https://www.hcup-us.ahrq.gov.

This retrospective cohort study isolated data on women admitted for childbirth-associated breast and nipple infections from the NIS between 2005–2014 using ICD-9 code 675. Various sub-diagnoses—namely 675.00, 675.10, and 675.20—corresponding to infections of the nipple associated with childbirth, abscess of breast associated with childbirth, and non-purulent mastitis associated with childbirth, respectively, were included. Specific codes 675.80 and 675.90, indicating other specified and unspecified infections of the breast and nipple associated with childbirth, were excluded. Women admitted for non-childbirth-related breast infections were also excluded from this study.

Datapoints accessible through the NIS database during inpatient stay were collected. This included primary diagnosis on admission, age, race, income quartile, and health care insurance, namely private insurance, Medicare, Medicaid, self-paid, no charge, or other. The severity of breast infection was determined to correlate to loss of function, i.e., the extent of physiologic decompensation of the breast tissue. This ranged from mild loss of function, defined as subclinical loss of lactation, to extreme loss of function, which corresponds to a functional mastotomy requiring abscess drainage. Type of admission (elective or emergent), patient comorbidities, hospital location (urban or rural), occurrence of a hospital procedure, time to first procedure, total charges, and hospital length of stay were compared. 

Statistical analysis was performed using SPSS, version 26. Elements were stratified by abscess, nipple infection, and mastitis; elective or emergency admission; and procedure or no procedure. The differences were examined using chi-square tests and *t*-tests. Backward multivariable linear regression assessed risk factors associated with hospital length of stay. Statistical significance was obtained at *p* < 0.05.

This study was exempt from Institutional Review Board (IRB) review as per guidelines put forth by our institutional IRB for analysis of HCUP databases. This study was conducted in compliance with the ethical standards of the responsible institution on human subjects as well as with the Helsinki Declaration.

## 3. Results

A total of 4614 patients were hospitalized with a primary diagnosis of breast infections associated with childbirth in the United States between 2005 and 2014 and compared against a total of 9,121,337 admissions for childbirth during this period (Table 1).

The overall rate of hospitalization for these infections is 0.05% within the general postpartum population. White women were hospitalized for breast infections at higher rates compared with national rates of childbirth in white women without breast infection. All other races had lower rates of hospitalized breast infections.

Of 4614 patients admitted with the primary diagnosis of a breast infection associated with childbirth, Table 2 stratifies the patients by type of primary diagnosis. Mastitis was the most common breast infection, followed by abscess and nipple infection.

The 22–30-year age group had the highest proportion of all breast diagnoses compared to all other groups, with a mean age range between 26.15 to 27.23 across the three primary diagnoses groups. This was followed, in decreasing proportion, by the age greater than 31, 18–21, and 12–17 age groups, respectively. White women experienced breast infections at the highest rates, accounting for more than half of all the reported cases of nipple infection and mastitis and nearly half of all cases of breast abscess. Black women had the second highest proportion of reported cases of nipple infection and mastitis and third highest for abscess, while Hispanic women were second in proportion for breast abscess and third for nipple infection and mastitis. Asian/Pacific Islander, other, and Native American, in descending order, together accounted for the remaining number of cases. There is a disproportionate difference in the breast diagnosis between White and Black women. In White women, the majority of admissions were for mastitis compared with abscess, 65.1% and 33.9%, respectively. In Black women, 60.0% of admissions were due to abscess, and 37.9% were due to mastitis. 

Patient reported income was stratified into four quartiles. At the conclusion of data collection in 2014, the quartiles were as follows: (1) USD 1–USD 39,999; (2) USD 40,000–USD 50,999; (3) USD 51,000–USD 65,999; and (4) USD 66,000+ [15]. Breast abscess was observed with decreasing frequency as income quartile increased. This was not the case for nipple infection, where the incidence in the fourth income quartile was higher than seen in the third. Regarding mastitis, more than half of cases were seen in the third and fourth income quartiles. Patients utilizing private insurance and Medicaid accounted for more than 90% of all cases. Most patients presenting with abscess and nipple infection had Medicaid as compared with the majority of patients with mastitis, who had private insurance.

An overwhelming majority of cases, independent of primary diagnosis, resulted in either minor or moderate loss of lactation. However, breast abscesses were associated with a greater degree of loss of lactation compared with nipple infection and mastitis. Major or extreme loss of function, defined as need for abscess drainage, occurred in less than one-tenth of cases. Several comorbidities were statistically significant in contributing to the incidence of mastitis and breast abscess. Diabetes, drug abuse, hypertension, obesity, and psychosis were most frequently associated with cases of breast abscess, whereas fluid and electrolyte disorders were the most common comorbidities in cases of mastitis. In cases with a primary nipple infection diagnosis, no comorbidities were found to be statistically significant.

Patients presenting with breast abscess were more likely to have a procedure, on average, within one day of admission. The data were not stratified by type of procedure, and both breasts, obstetrics, and other procedures are included. These patients were also more likely to have a longer hospitalization, averaging 3.34 days, compared with an average 2.29-day admission for nipple infection and 2.49-day admission for mastitis. Patients admitted with breast abscess were charged the highest, an average of USD 17,509, followed in descending proportion by nipple infection and mastitis.

In a second analysis, patients were stratified into two groups by admission type, namely elective, scheduled admissions, and emergency admissions, with 748 cases and 3851 cases, respectively, for each group (Table 3). Stratifying our data based on admission type provided additional insight into the severity of infection at presentation.

There were differences noted for numerous variables regarding admission type but not for age or income quartile. The average age was 26 years, and admissions were evenly distributed throughout the four income quartiles. Across all cases, a higher percentage of White women were electively admitted as compared to Black, Hispanic, and Asian/Pacific Islander women, who made up a greater percentage of emergency admissions. Of note, in Black women, the proportion of emergency admission was nearly double that of elective admissions. Private insurance, followed by Medicaid, was most used for emergency admissions, although private insurance made up a larger percentage of elective admissions and Medicaid a larger percentage of emergency admission.

The primary diagnoses of breast abscess, nipple infection, and mastitis occurred at the same rates in both elective and emergency breast-related admissions. The distribution showed mastitis was most common (57.9–58.7%), followed by breast abscess (40.6%) and infections of the nipple (0.6–1.5%). In greater than 90% of cases, patients experienced minor or moderate loss of lactation. However, the percentage of minor loss was greater in the elective admission group, whereas the rate of moderate, major, and severe loss of function, including the need for abscess drainage, was higher in the emergency admission group. Notably, no patients electively admitted required drainage.

Relevant comorbidities included coagulopathy, which was more prevalent in the elective admission group, and deficiency anemia, chronic blood loss, and fluid and electrolyte abnormalities, which were more prevalent in the emergency admission group. Length of stay, occurrence of breast procedure, and time to procedure were comparable between the two groups. Total charges varied significantly, with emergency admissions totaling approximately USD 3000 more than their elective counterparts.

The association between hospital LOS and various factors was analyzed using backward linear regression analysis (Table 4). Primary breast diagnosis (abscess, nipple infection, or mastitis), hospital type (urban teaching, urban non-teaching, or rural), application of invasive diagnostic procedures, status post-surgical-procedure status or operation such as caesarian delivery, severity of functional loss, and various comorbidities were significantly associated with increased LOS.

A primary breast diagnosis of abscess was significantly correlated to increased LOS as compared with a diagnosis of mastitis, which did not increase LOS. Urban teaching and urban non-teaching hospitals observed significantly longer LOS in those with breast abscess or breast infection, but this connection was not found in rural hospitals. Invasive diagnostic procedures, surgical procedures, and cesarean sections were all associated with prolonged LOS. Undergoing a surgical procedure showed the strongest correlation between these three variables. All severities of functional loss, ranging from loss of lactation (mild) to abscess drainage (severe), were related to prolonged hospitalizations, and the comorbidities most correlated to increased LOS were deficiency anemias, obesity, and neurological disorders.

Table 5 presents the study population stratified by various characteristics and the presence of a hospital procedure. Procedures include breast-specific procedures such as biopsy, incision and drainage, aspiration, excision, and suture as well as non-breast-related procedures such as diagnostic imaging, catheter placement, injections and infusions of treatment, and non-breast surgical procedures. Overall, 47.8% of patients underwent a procedure, and 52.2% did not. Of those who had a hospital procedure, 83% had a breast-related procedure, and 17% had a non-breast-related procedure. Further stratification by primary diagnosis showed that of the patients who underwent a breast-related procedure, 84.4% were diagnosed with a breast abscess compared with 15.3% who had mastitis and 0.03% who had a nipple infection. Of the 1873 patients who presented with a breast abscess, 82.1% (1541) had a procedure. Of the 2703 patient who had mastitis, 10.43% (279) had a procedure.

Further analysis showed significant differences among rates of breast procedure based on race, income, insurance, age, total charges, and length of stay. Undergoing a breast procedure such as biopsy, incision and drainage, aspiration, and excision is associated with greater functional loss and higher disease severity. In terms of race, 57.8% of Black women underwent a breast procedure compared to 34.3% of White women and 42.6% of Hispanic women. Pacific Islander, Native American, and other women underwent breast procedures at comparable rates ranging between 40–50%. For income, rates of procedure decreased as income quartile increased. The highest rate of breast procedure, 47.9%, occurred in the lowest quartile. Rates of breast procedure were also higher in the Medicare and Medicaid groups, at 48.1% and 46.7%, respectively, compared to the rate of 33% in women with private insurance. The mean age of patients with a breast procedure was 26.17 years, those with a non-breast procedure was 26.96 years, and those with no procedure was 27.15 years. Total charges were highest in the breast-procedure group, averaging USD 18,856 compared to an average of USD 16,040 in the non-breast-procedure group and USD 8902 in the no-procedure group. Lastly, the length of stay was significantly longer, with an average of 3.52 days in the breast-procedure group, followed by 2.93 days in the non-breast-procedure group and 2.29 days in the no-procedure group.

## 4. Discussion

The primary aim of this study was to evaluate risk factors strongly associated with breast and nipple infections related to childbirth in hospitalized women, with special consideration of factors influencing hospital length of stay. Age, race, and socioeconomic factors were found to be predictive of hospitalization for breast infection, while primary diagnosis, hospital procedure, severity of functional loss, and the comorbidities of anemia, diabetes, drug abuse, hypertension, obesity, and psychosis were significantly associated with increased hospital LOS. These results represent novel findings, as they reflect comprehensive underlying factors including demographics, socioeconomic status, and medical history as predictors of outcomes for management of peripartum breast infection, whereas the current literature focuses on factors related to lactation and breastfeeding practices.

The mean age of patients was observed to be 26.75 years, with the highest frequency distribution of cases in patients aged 22–30 years. This age corresponds to the average age of first birth in the United States, determined to be a mean of 26.3 years in a national sample from 2014 [16]. Previous studies have shown inconsistent findings identifying age as a risk factor for lactational infections; however, others have suggested that primiparous women experience lactational mastitis and nipple infection at higher rates [17,18,19,20]. The hypothesis we propose as a potential cause of childbirth-associated breast infections is poor breastfeeding practices in first-time mothers. Difficulty breastfeeding, problems with latching, cracked and sore nipples, and improper nursing technique increase risk of breast infections most commonly in first-time nursing mothers [17,21,22,23]. This suggests that increased lactation education and resources may be beneficial for mothers choosing to breastfeed following their first birth.

Two of the most significant risk factors for increased hospital length of stay were a primary diagnosis of breast abscess, which is a potential complication of both mastitis and nipple infection [5,6], and presence of a surgical procedure, invasive diagnostic procedure, and cesarian section. Rates of breast abscess were also higher in women undergoing a surgical procedure, including caesarean delivery. This finding is supported in the literature identifying cesarean section and delivery in a hospital as predictive risk factors for breast infection due to suboptimal breastfeeding practices caused by increased separation between the mother and newborn and inconsistent breastfeeding times [22,23]. It is known that most U.S. hospitals do not allow room-sharing between mothers and newborns following delivery. Cesarean delivery is especially linked with delayed skin-to-skin contact, further separation of mother and baby, and increased supplementary feeding, which further contributes to poor breastfeeding practices and increased rates of infection and its potential complications [24].

Regarding hospital location, prolonged hospitalizations were more strongly associated with both urban teaching and urban non-teaching hospitals as compared to rural hospitals. Although no current literature has studied the differences in urban versus rural hospitalizations for childbirth-associated breast infections, this finding is consistent with the current overall trend in the United States. According to the CDC National Center for Health Statistics, HLOS is on average longer in urban hospitals. This in part may be because those at urban hospitals undergo more procedures, or those with more complicated disease courses are transferred to tertiary or quaternary care hospitals in an urban setting [25]. This former presumption would be consistent with our findings, as we have shown that HLOS was strongly associated with undergoing invasive diagnostic procedures and having an obstetric procedure. 

Additional predictive factors were socioeconomic considerations. Women in lower-income quartiles or with Medicaid insurance were more likely to be admitted with a breast abscess after mastitis and had a higher severity of illness on average. They were also more likely to need an emergency admission, require a breast procedure, and have a prolonged hospital length of stay. Although we cannot determine whether this is due to lack of patient education, lack of outpatient treatment options, or other factors, this finding supports socioeconomic factors as contributing to infection course and poorer outcomes. 

Further observations include the difference in proportion of mastitis and breast abscess in White women compared to Black women. As breast abscess is a progression of mastitis, often due to delayed or absent treatment, this indicates deficiency in the management of mastitis. This could be attributed to poorer management in Black women, whether due confounding variables related to barriers to care, such as insurance or income, or inherent to race itself, potentially due to delayed recognition of mastitis in women of color [26]. In addition, lack of diverse representation in medical textbooks and the literature has made it challenging for physicians to identify certain disorders, especially dermatologic disorders, in people of color. Given that mastitis is most often a clinical diagnosis, and there are differences in appearance contingent on skin tone, the rates of timely diagnosis and treatment may vary between races [27,28,29,30]. This is especially important considering findings that Black women undergo breast procedures at higher rates, which is associated with higher severity of functional loss, higher total hospital charges, and longer hospital stays. 

Comorbidities considerably related to breast abscess were found to be obesity, hypertension, diabetes, and drug abuse, which are consistent with the current literature [31]. Although obesity, defined as BMI >30, and diabetes are known to be associated with development of breast abscess, recurrent abscess, and increased hospital length of stay, hypertension and drug abuse have shown mixed results [32].

A major strength of analysis of a widespread sample size via the National Inpatient Sample, containing data on patients across the United States over a ten-year period, allows for the generalizability of this study, with applicability to women with primary breast and nipple infections associated with childbirth. We identified patterns and established potential additional risk factors leading to mastitis and progression to breast abscess. Our findings strongly support using type of diagnosis, occurrence of hospital procedure, socioeconomic considerations, and comorbidities to identify patients most at risk for breast infection, poorer breastfeeding outcomes, and increased hospital length of stay.

This analysis is not without limitations, as there is inherent weakness in large database analysis. The NIS has a data structure such that each observation represents a discrete health care encounter and includes a set of administrative diagnosis and procedure codes that correspond with that encounter; thus, there is no ability to track patients longitudinally. NIS does not provide all information regarding patient medical history and demographics, diagnostic criteria, treatment, or outpatient follow-up and outcomes and also does not always explicitly define its data points (i.e., further definition of functional loss); thus, we realize this as a limitation of our study. Therefore, certain datapoints may be missing for some individuals. In addition, breast infections can be managed successfully with outpatient strategy and treatment; however, patient self-reported data on breastfeeding practices as well as treatment and outcomes independent of the hospitalizations are not available. Given that our data are limited to patients in the inpatient setting, our study cannot be applied to women who undergo outpatient treatment of a breast infection. This is a limitation of large database review, whereby the data available are not granular in regard to establishing cause and effect.

Further, the NIS study population includes women admitted with a primary diagnosis of childbirth-associated breast infections and not women admitted for childbirth. Therefore, we surmise that cesarean sections occurred prior to the current hospitalization for the breast infection. However, we cannot know whether other surgical or diagnostic procedures occurred during this current hospitalization or prior hospitalizations.

Similarly, as a major finding in this analysis was occurrence of hospital procedure increasing the length of stay, additional information on the timeline of diagnosis and procedure as well as the initial course of treatment, if any, could prove useful, including outpatient antibiotic treatment or prior breast abscess drainage. The addition of such data would considerably enhance our current understanding and further data stratification. Further, analyses of large databases do not allow cross-correlation to identify confounding risk factors. For example, data showing a variation in diagnosis or outcomes among the various racial groups may be connected to race alone, when the true correlation is due to a linked variable such as income quartile or type of insurance. A final limitation is the exclusion of patients with childbirth-associated breast and nipple infections who did not seek medical attention or were not admitted to the hospital. Therefore, our findings cannot be applied to women who elect for outpatient management of breast abscess, and risk factors and overall disease burden may be biased.

Further studies should examine these findings, as they relate to breastfeeding practices and concentrate on establishing best practices for risk reduction and prevention. We especially believe a prospective long-term analysis of outcomes beyond hospitalization and impact on breastfeeding practices would be beneficial to our understanding of breast infections associated with childbirth.

## 5. Conclusions

In conclusion, primary diagnosis of breast abscess and occurrence of hospital procedure were most significantly associated with prolonged LOS in women diagnosed with breast and nipple infections associated with childbirth. Factors such as age, socioeconomic position, severity of functional loss, as well as comorbidities were also contributing risk factors to the development of breast infection and increased hospital LOS. Further studies should examine these findings, as they relate to breastfeeding practices and concentrate on establishing best practices for risk reduction and prevention of childbirth-associated breast and nipple infections and hospitalizations.

## Figures and Tables

**Table 1:** Patient characteristics with diagnosis of breast infections associated with childbirth, compared with all other deliveries in the U.S. NIS 2005–2014.

		The Current Study Population, N (%)	All Other Deliveries, N (%)	*p*
All Cases		4614	9,116,723	
**Race**	**White**	2157 (55.05%)	3,953,401 (51.7%)	0.003
	**Black**	535 (13.65%)	1,120,382 (14.6%)	
	**Hispanic**	856 (21.85%)	1,760,773 (23%)	
	**Asian/Pacific Islander**	159 (4.05%)	380,928 (5%)	
	**Native American**	30 (0.8%)	64,059 (0.8%)	
	**Other**	34 (4.6%)	370,205 (4.8%)	
**Income** **Quartile**	**Quartile 1**	1279 (28%)	2,501,886 (28%)	0.007
	**Quartile 2**	1064 (23.4%)	2,271,203 (25.4%)	
	**Quartile 3**	1145 (25.25%)	2,192,311 (24.5%)	
	**Quartile 4**	1067 (23.35%)	1,980,779 (22.1%)	
**Insurance**	**Private Insurance**	2309 (50%)	4,480,575 (49.2%)	0.001
	**Medicare**	27 (0.6%)	68,482 (0.8%)	
	**Medicaid**	1931 (42%)	3,968,701 (43.6%)	
	**Self-Pay**	186 (4%)	315,717 (3.5%)	
	**No Charge**	19 (0.4%)	19,080 (0.2%)	
	**Other**	138 (3%)	254,963 (2.8%)	
**Continuous Variables**		**Mean (SD)**	**Mean (SD)**	***p***
**Age, Years**		26.75 (6)	27.7 (6.1)	0.0001
**Time to First Procedure, Days**		0.93 (1.25)	0.47 (2.08)	0.0001
**Length of Stay, Days**		2.83 (1.9)	2.67 (2.64)	0.0001
**Total Charges, Dollars**		13,407 (12,783)	13,115 (15,065)	0.5

**Table 2:** Patient characteristics with diagnosis of breast infections associated with childbirth, stratified according to the primary diagnosis. NIS 2005–2014.

		Abscess, N (%)	Nipple Infection, N (%)	Mastitis, N (%)	*p*
All Cases		1876 (40.7%)	35 (0.8%)	2703 (58.6%)	
**Age Group**	**12–17 years**	114 (6.1%)	1 (2.9%)	97 (3.6%)	**<0.001**
	**18–21 years**	350 (18.7%)	7 (20.0%)	423 (15.7%)	
	**22–30 years**	942 (50.4%)	15 (42.9%)	1342 (49.7%)	
	**31+ years**	464 (24.8%)	12 (34.3%)	838 (31.0%)	
**Race**	**White**	733 (45.4%)	19 (57.6%)	1405 (61.9%)	**<0.001**
	**Black**	325 (20.1%)	7 (21.2%)	203 (8.9%)	
	**Hispanic**	393 (24.3%)	4 (12.1%)	459 (20.2%)	
	**Asian/Pacific Islander**	68 (4.2%)	1 (3.0%)	90 (4.0%)	
	**Native American**	14 (0.9%)	0 (0%)	16 (0.7%)	
	**Other**	81 (5.0%)	2 (6.1%)	98 (4.3%)	
**Income** **Quartile**	**Quartile 1**	620 (33.6%)	11 (31.4%)	648 (24.2%)	**<0.001**
	**Quartile 2**	439 (23.8%)	11 (31.4%)	614 (22.9%)	
	**Quartile 3**	422 (22.9%)	5 (14.3%)	718 (26.8%)	
	**Quartile 4**	363 (19.7%)	8 (22.9%)	696 (26.0%)	
**Insurance**	**Private Insurance**	749 (40.0%)	13 (37.1%)	1547 (57.3%)	**<0.001**
	**Medicare**	14 (0.7%)	0 (0%)	13 (0.5%)	
	**Medicaid**	958 (51.1%)	19 (54.3%)	954 (35.3%)	
	**Self-Pay**	95 (5.1%)	2 (5.7%)	89 (3.3%)	
	**No Charge**	12 (0.6%)	1 (2.9%)	6 (0.2%)	
	**Other**	46 (2.5%)	0 (0%)	92 (3.4%)	
**Severity**	**Minor Loss of Function**	278 (14.8%)	25 (71.4%)	1716 (63.5%)	**<0.001**
	**Moderate Loss of Function**	1524 (81.2%)	7 (20.0%)	789 (29.2%)	
	**Major Loss of Function**	67 (3.6%)	3 (8.6%)	186 (6.9%)	
	**Extreme Loss of Function**	7 (0.4%)	0 (0%)	12 (0.4%)	
**Comorbidities**	**AIDS**	2 (0.1%)	0 (0%)	0 (0%)	0.230
	**Alcohol Abuse**	4 (0.2%)	0 (0%)	0 (0%)	0.054
	**Deficiency Anemias**	149 (7.9%)	3 (8.6%)	170 (6.3%)	0.090
	**Rheumatoid Arthritis**	8 (0.4%)	0 (0%)	10 (0.4%)	0.890
	**Chronic Blood Loss**	132 (7.0%)	3 (8.6%)	169 (6.3%)	0.510
	**Congestive Heart Failure**	0 (0%)	0 (0%)	1 (0.0%)	0.700
	**Chronic Pulmonary Disease**	81 (4.3%)	1 (2.9%)	106 (3.9%)	0.750
	**Coagulopathy**	8 (0.4%)	1 (2.9%)	15 (0.6%)	0.130
	**Depression**	53 (2.8%)	0 (0%)	70 (2.6%)	0.550
	**Diabetes, Uncomplicated**	34 (1.8%)	0 (0%)	27 (1.0%)	**0.048**
	**Diabetes, Chronic Complications**	9 (0.5%)	0 (0%)	1 (0.0%)	**0.006**
	**Drug Abuse**	29 (1.5%)	1 (2.9%)	6 (0.2%)	**<0.001**
	**Hypertension**	54 (2.9%)	0 (0%)	41 (1.5%)	**0.004**
	**Hypothyroidism**	29 (1.5%)	0 (0%)	62 (2.3%)	0.140
	**Liver Disease**	4 (0.2%)	0 (0%)	4 (0.1%)	0.850
	**Lymphoma**	2 (0.1%)	0 (0%)	0 (0%)	0.230
	**Fluid/Electrolyte Disorders**	81 (4.3%)	1 (2.9%)	274 (10.1%)	**<0.001**
	**Other Neurological Disorders**	10 (0.5%)	0 (0%)	16 (0.6%)	0.870
	**Obesity**	67 (3.6%)	1 (2.9%)	25 (0.9%)	**<0.001**
	**Psychoses**	27 (1.4%)	2 (5.7%)	11 (0.4%)	**<0.001**
	**Pulmonary Circulation Disorders**	0 (0%)	0 (0%)	2 (0.1%)	0.490
	**Renal Failure**	2 (0.1%)	0 (0%)	2 (0.1%)	0.920
	**Solid Tumor**	1 (0.1%)	0 (0%)	2 (0.1%)	0.950
	**Valvular Disease**	3 (0.2%)	0 (0%)	12 (0.4%)	0.240
	**Weight Loss**	3 (0.2%)	0 (0%)	6 (0.2%)	0.870
**Emergency Admission**		1565 (83.7%)	24 (68.6%)	2262 (83.9%)	**0.050**
**Had a Procedure**		1533 (81.7%)	9 (25.7%)	255 (9.4%)	**<0.001**
		**Mean (SD)**	**Mean (SD)**	**Mean (SD)**	***p***
**Age, Years**		26.15 (6.14)	27.23 (6.05)	27.15 (5.94)	**<0.001**
**Time to First Procedure, Days**		0.85 (1.20)	1.00 (0.76)	1.39 (1.57)	**<0.001**
**Length of Stay, Days**		3.34 (2.37)	2.29 (1.34)	2.49 (1.52)	**<0.001**
**Total Charges, Dollars**		17,509 (15,506)	11,695 (8743)	10,554 (10,811)	**<0.001**

**Table 3:** Characteristics of women admitted with breast infections associated with childbirth, stratified as emergent vs. elective admission. NIS 2005–2014.

		Elective, N (%)	Emergency, N (%)	*p*
All Cases		748 (16.3%)	3851 (83.7%)	
**Age Group**	**12–17 years**	39 (5.3%)	173 (4.5%)	0.220
	**18–21 years**	108 (14.4%)	669 (17.4%)	
	**22–30 years**	381 (51.0%)	1911 (49.7%)	
	**31+ years**	220 (29.3%)	1089 (28.4%)	
**Race**	**White**	394 (65.6%)	1756 (53.2%)	**<0.001**
	**Black**	50 (8.3%)	485 (14.7%)	
	**Hispanic**	98 (16.2%)	754 (22.8%)	
	**Asian/Pacific Islander**	21 (3.5%)	138 (4.2%)	
	**Native American**	5 (0.8%)	25 (0.8%)	
	**Other**	34 (5.6%)	146 (4.4%)	
**Income** **Quartile**	**Quartile 1**	221 (29.7%)	1052 (27.6%)	0.080
	**Quartile 2**	190 (25.9%)	872 (23.0%)	
	**Quartile 3**	162 (21.7%)	980 (25.8%)	
	**Quartile 4**	169 (22.7%)	895 (23.6%)	
**Insurance**	**Private Insurance**	413 (55.5%)	1887 (49.0%)	**0.003**
	**Medicare**	3 (0.4%)	23 (0.6%)	
	**Medicaid**	275 (36.7%)	1652 (42.9%)	
	**Self-Pay**	21 (2.8%)	165 (4.3%)	
	**No Charge**	3 (0.4%)	16 (0.4%)	
	**Other**	31 (4.1%)	107 (2.8%)	
**Breast** **Diagnosis**	**Abscess of Breast**	304 (40.6%)	1565 (40.6%)	**0.050**
	**Infections of Nipple**	11 (1.5%)	24 (0.6%)	
	**Nonpuerperal Mastitis**	433 (57.9%)	2262 (58.7%)	
**Severity**	**Minor Loss of Function**	365 (48.6%)	1652 (43.0%)	**0.011**
	**Moderate Loss of Function**	351 (46.7%)	1959 (50.8%)	
	**Major Loss of Function**	35 (4.7%)	222 (5.7%)	
	**Extreme Loss of Function**	0 (0%)	19 (0.5%)	
**Comorbidities**	**AIDS**	0 (0%)	2 (0.1%)	0.999
	**Alcohol Abuse**	1 (0.1%)	3 (0.1%)	0.510
	**Deficiency Anemias**	37 (4.9%)	284 (7.4%)	**0.017**
	**Rheumatoid Arthritis**	3 (0.4%)	15 (0.4%)	0.999
	**Chronic Blood Loss**	37 (4.9%)	266 (6.9%)	**0.048**
	**Congestive Heart Failure**	0 (0%)	1 (0.0%)	0.999
	**Chronic Pulmonary Disease**	21 (2.8%)	167 (4.3%)	0.053
	**Coagulopathy**	8 (1.1%)	16 (0.4%)	**0.023**
	**Depression**	14 (1.9%)	109 (2.8%)	0.140
	**Diabetes, Uncomplicated**	7 (0.9%)	54 (1.4%)	0.310
	**Diabetes, Chronic Complications**	0 (0%)	10 (0.3%)	0.380
	**Drug Abuse**	5 (0.7%)	31 (0.8%)	0.700
	**Hypertension**	13 (1.7%)	81 (2.1%)	0.520
	**Hypothyroidism**	13 (1.7%)	77 (2.0%)	0.640
	**Liver Disease**	1 (0.1%)	7 (0.2%)	0.999
	**Lymphoma**	1 (0.1%)	0 (0.0%)	0.160
	**Fluid/Electrolyte Disorders**	37 (4.9%)	318 (8.3%)	**0.002**
	**Other Neurological Disorders**	3 (0.4%)	23 (0.6%)	0.790
	**Obesity**	12 (1.6%)	81 (2.1%)	0.380
	**Psychoses**	6 (0.8%)	34 (0.9%)	0.830
	**Pulmonary Circulation Disorders**	0 (0%)	2 (0.1%)	0.999
	**Renal Failure**	0 (0%)	4 (0.1%)	0.999
	**Solid Tumor**	1 (0.1%)	2 (0.1%)	0.410
	**Valvular Disease**	3 (0.4%)	12 (0.3%)	0.720
	**Weight Loss**	3 (0.4%)	6 (0.2%)	0.170
**Had a Procedure**		275 (36.8%)	1516 (39.4%)	0.180
		**Mean (SD)**	**Mean (SD)**	***p***
**Age, Years**		26.86 (6.01)	26.72 (6.05)	0.580
**Time to First Procedure, Days**		0.82 (1.15)	0.94 (1.29)	0.150
**Length of Stay, Days**		2.77 (1.84)	2.84 (1.98)	0.350
**Total Charges, Dollars**		10,677 (10,483)	13,930 (13,801)	**<0.001**

**Table 4:** Backward linear regression analysis to evaluate associations between LOS and factors in women with a primary diagnosis of infection of the breast and nipple associated with childbirth. Top 5% hospital length of stays were removed prior to analysis. NIS 2005–2014.

		Multivariable Linear Regression (HLOS)	
		N = 4387	*p*
		β (95% CI)	
**Intercept**		2.051 (1.981, 2.121)	**<0.001**
**Hospital** **Location**	**Urban: Teaching**		**0.019**
	**Rural**	0.100 (–0.029, 0.230)	0.130
	**Urban: Non-Teaching**	0.112 (0.030, 0.193)	**0.007**
**Breast** **Diagnosis**	**Abscess**		**0.002**
	**Infection**	–0.201 (–0.322, –0.079)	**0.001**
	**Mastitis**	–0.387 (–0.817, 0.043)	0.080
**Invasive Diagnostic Procedure**		0.364 (0.112, 0.617)	**0.005**
**Surgical Procedure**		0.713 (0.602, 0.824)	**<0.001**
**Cesarean Section**		1.158 (0.203, 2.113)	**0.017**
**Severity**	**Minor Loss of Function**		**<0.001**
	**Moderate Loss of Function**	0.292 (0.200, 0.384)	**<0.001**
	**Major Loss of Function**	0.603 (0.426, 0.780)	**<0.001**
	**Extreme Loss of Function**	0.766 (0.134, 1.398)	**0.018**
**Comorbidities**	**Deficiency Anemias**	0.385 (0.231, 0.539)	**<0.001**
	**Drug Abuse**	–0.784 (–1.254, –0.314)	**0.001**
	**Other Neurological Disorders**	0.522 (0.007, 1.037)	**0.047**
	**Obesity**	0.334 (0.052, 0.616)	**0.020**
**Age, Years**		**Removed** **Via** **Backward** **Elimination**	
**Race**			
**Income Quartile**			
**Insurance**			
**Comorbidities**	**AIDS**		
	**Alcohol Abuse**		
	**Rheumatoid Arthritis**		
	**Chronic Blood Loss**		
	**Congestive Heart Failure**		
	**Chronic Pulmonary Disease**		
	**Coagulopathy**		
	**Depression**		
	**Diabetes, Uncomplicated**		
	**Diabetes, Chronic Complications**		
	**Hypertension**		
	**Hypothyroidism**		
	**Liver Disease**		
	**Lymphoma**		
	**Fluid/Electrolyte Disorders**		
	**Psychoses**		
	**Pulmonary Circulation Disorders**		
	**Renal Failure**		
	**Solid Tumor**		
	**Valvular Disease**		
	**Weight Loss**		

**Table 5:** Characteristics of women with primary diagnosis of infections of the breast and nipple associated with childbirth, stratified by breast procedures, non-breast procedure, or no procedure. NIS 2004–2014.

		No Procedure	Breast Procedure	Non-Breast Procedure	*p*
		N (%)			
**Primary Diagnosis**	Breast Abscess	235 (12.5%)	1541 (82.1%)	100 (5.3%)	**<0.001**
	Mastitis	2157 (79.8%)	279 (10.3%)	267 (9.9%)	
	Nipple Infection	6 (31.6%)	6 (31.6%)	7 (36.8%)	
**Race**	White	1232 (56.9%)	743 (34.3%)	192 (8.9%)	**<0.001**
	Black	187 (34.9%)	310 (57.8%)	39 (7.3%)	
	Hispanic	411 (47.8%)	366 (42.6%)	82 (9.5%)	
	Asian/Pacific Islander	80 (50%)	65 (40.6%)	15 (9.4%)	
	Native American	15 (50%)	15 (50%)	0 (0.0%)	
	Other	82 (45.3%)	78 (43.1%)	21 (11.6%)	
**Income Quartile**	Quartile 1	566 (44.2%)	614 (47.9%)	101 (7.9%)	**<0.001**
	Quartile 2	569 (53.1%)	414 (38.7%)	88 (8.2%)	
	Quartile 3	630 (54.7%)	414 (36%)	107 (9.3%)	
	Quartile 4	619 (57.8%)	356 (33.2%)	96 (9%)	
**Insurance**	Private Insurance	1364 (58.8%)	764 (33%)	190 (8.2%)	**<0.001**
	Medicare	14 (51.9%)	13 (48.1%)	0 (0%)	
	Medicaid	848 (43.8%)	904 (46.7%)	185 (9.6%)	
	Self-Pay	84 (44.4%)	93 (49.2%)	12 (6.3%)	
	No Charge	7 (36.8%)	11 (57.9%)	1 (5.3%)	
	Other	87 (62.6%)	41 (29.5%)	11 (7.9%)	
		**Mean (SD)**			***p***
**Age, Years**		27.15 (5.96)	26.17 (6.17)	26.96 (5.80)	**<0.001**
**Total Charges**		8902 (7072)	18,856 (16,956)	16,040 (14,213	**<0.001**
**Length of Stay**		2.29 (1.13)	3.52 (2.46)	2.93 (1.89)	**<0.001**
**Days from Admission to First Procedure**		0.33 (0.5)	0.97 (1.43)	0.86 (1.48)	0.196

## Data Availability

The National Inpatient Sample dataset, a part of the Healthcare Cost and Utilization Project sponsored by the Agency for Healthcare Research and Quality, is available from the corresponding author on reasonable request.
